# Mechanisms by which varicella zoster virus contributes to Alzheimer’s disease pathologies

**DOI:** 10.1002/alz.090837

**Published:** 2025-01-09

**Authors:** Maria A. Nagel, Andrew N. Bubak, Niklaus H. Mueller, Christy S. Niemeyer, Ravi Mahalingam

**Affiliations:** ^1^ University of Colorado School of Medicine, Aurora, CO USA

## Abstract

**Background:**

Varicella zoster virus (VZV) reactivation, manifesting as herpes zoster, increases dementia risk. Herein, we review the literature supporting the biological plausibility of VZV contributing to AD pathologies and examine the unique ability of VZV to induce amylin that has been found in blood vessels and parenchyma of AD patients.

**Method:**

We conducted a literature review on VZV and dementia to elucidate a potential model for how VZV reactivation intersects with AD. Further, we cloned the human amylin promoter into a luciferase reporter plasmid that was transfected into primary human brain vascular adventitial fibroblasts. Cells were then infected with multiple strains of VZV (Gilden, vOka) and HSV‐1 (McKrae, Kos) or transfected with plasmids expressing individual VZV or HSV‐1 proteins. Luciferase activity was subsequently measured.

**Result:**

After reactivation, VZV has been shown to infect cerebral arteries, produce a similar spectrum of cerebrovascular disease (VZV vasculopathy) similar to that seen in AD, and cause long‐lasting cognitive impairment. Importantly, we found VZV‐infected primary human brain vascular adventitial fibroblasts (HBVAFs) contain intracellular amyloidogenic peptides seen in AD plaques (amyloid‐beta (Ab)‐42, amylin) and amyloid; RNA sequencing showed enrichment of pathways of amyloidosis/AD, with upstream regulators amyloid precursor protein (APP), apolipoprotein E, microtubule‐associated protein tau, presenilin 1, and amylin (IAPP). VZV vasculopathy patient cerebrospinal fluid (CSF) is amyloidogenic, has elevated amyloid and amylin (correlating with anti‐VZV antibody titers), and decreased Ab‐40 that is seen in cerebral amyloid angiopathy (CAA). VZV antigen/DNA co‐localized with amyloid‐containing arteries in select CAA cases. Finally, we found that VZV infection, but not HSV‐1 infection, induces luciferase activity from the amylin promoter and identified a specific immediate early VZV protein that was sufficient to activate the amylin promoter.

**Conclusion:**

Our studies support the notion that VZV may contribute to AD progression, in part through infection of cerebral arteries, that leads to amylin expression and amyloid deposition. This VZV vasculopathy likely accounts for a subset, if not all, CAA seen in AD brains and is consistent with a previous study linking VZV to vascular dementia.

